# Improved survival prognostication of node-positive malignant melanoma patients utilizing shotgun proteomics guided by histopathological characterization and genomic data

**DOI:** 10.1038/s41598-019-41625-z

**Published:** 2019-03-26

**Authors:** Lazaro Hiram Betancourt, Krzysztof Pawłowski, Jonatan Eriksson, A. Marcell Szasz, Shamik Mitra, Indira Pla, Charlotte Welinder, Henrik Ekedahl, Per Broberg, Roger Appelqvist, Maria Yakovleva, Yutaka Sugihara, Kenichi Miharada, Christian Ingvar, Lotta Lundgren, Bo Baldetorp, Håkan Olsson, Melinda Rezeli, Elisabet Wieslander, Peter Horvatovich, Johan Malm, Göran Jönsson, György Marko-Varga

**Affiliations:** 1 Lund University, Lund, Sweden; 2 Warsaw University of Life Sciences SGGW, Warszawa, Poland; 3 University of Groningen, Groningen, The Netherlands; 4 National Koranyi Institute of Pulmonology, Budapest, Hungary; 5 Semmelweis University, Budapest, Hungary; 6 Tokyo Medical University, Tokyo, Japan

## Abstract

Metastatic melanoma is one of the most common deadly cancers, and robust biomarkers are still needed, e.g. to predict survival and treatment efficiency. Here, protein expression analysis of one hundred eleven melanoma lymph node metastases using high resolution mass spectrometry is coupled with in-depth histopathology analysis, clinical data and genomics profiles. This broad view of protein expression allowed to identify novel candidate protein markers that improved prediction of survival in melanoma patients. Some of the prognostic proteins have not been reported in the context of melanoma before, and few of them exhibit unexpected relationship to survival, which likely reflects the limitations of current knowledge on melanoma and shows the potential of proteomics in clinical cancer research.

## Introduction

The incidence of malignant melanoma is increasing worldwide, particularly in Western countries, and survival does not seem to improve substantially^1^. Primary surgery is curative in most patients but around 10–15% of tumors are showing progression. Thus, it is important to early identify those patients who carry a skin tumor with progressive pathobiology. Currently, Breslow thickness is the most accurate tool for predicting the disease outcome of primary melanoma^2^. To improve the prediction of disease outcome, more fine-tuned molecular profiling and data integration tools and efforts are needed to search for alternative biomarkers^3^.

Metastatic melanoma (MM) still remains a tumor with poor outcome^4,5^ despite interventions with targeted therapy and antibody-driven modulation of the immune response^6–11^.

Recent technological developments utilizing both genomic and proteomic analysis provide the opportunity to identify better predictive markers of melanomas^12–16^. It is possible to monitor the expression of certain genes and also gain understanding how these genes are expressed and regulated as functional proteins. Accordingly, detailed, personalized information on gene and protein expression and regulation, as well as data on specific mutations that may guide the treatment, can be monitored. Another cornerstone of prognostic predictions is clinicopathological characterization based on high quality pathological and clinical information. Equally important is to investigate the cellular composition of the tissue, to morphologically assess in detail the quality of tumor samples submitted for analysis and the identification of features important for disease progression.

In this study, we combine in depth histopathology analysis of melanoma lymph node metastases with deep-mining protein expression analysis using high-resolution mass spectrometry and a complex bioinformatics workflow to integrate clinical data with protein and genomics profile information. The protein data is matched to genomic analysis of the same tumor tissue. This information coupled with extensive clinical information on each subject provides an excellent opportunity to identify novel protein markers to predict progression and survival of melanoma.

## Results and Discussion

### Clinical data

A total of 111 patients diagnosed with melanoma metastasis between 1975 and 2011 were evaluated in the study (Table 1). There were 68 men and 43 women among the investigated cases. Average age ± standard deviation (range) at diagnosis of lymph node metastasis was 62.4 ± 13.7 (25–89) years. The time elapsed to progression from primary tumor to lymph node metastasis was 5.0 ± 5.6 (0–18.0) years and overall survival was 7.9 ± 6.8 (0.2–43.0) years. The dominant histotypes of primary tumors were Superficial Spreading Melanoma (SSN) and Nodular Melanoma (NM) (see Table 1). The cohort included 59% of patients with wild type BRAF status and 36% of patients with V600E mutation in the BRAF gene (4% had V600A or V600K mutation).

**Table 1:** Clinicopathological information about the patients and patient samples.

Clinicopathological properties		n	% of total
Gender	*Female*	43	39
	*Male*	68	61
Location	*trunk*	47	42
	*head/neck*	1	1
	*upper extremity*	12	11
	*lower extremity*	27	24
	*other*	7	6
Histological type	*SSM*	27	24
	*NM*	35	32
	*ALM*	4	4
	*LMM*	1	1
	*Mucosal*	1	1
	*Other*	1	1
	*Unknown*	13	12
Clark level	*1*	1	1
	*2*	4	4
	*3*	25	23
	*4*	43	39
	*5*	5	5
Breslow scale mm	*<1.00*	11	10
	*<2.00*	26	23
	*<3.00*	23	21
	*<4.00*	27	24
BRAF status	*V600E mut*	38	34
	*V600K mut*	3	3
	*V600A mut*	1	1
	*WT*	64	58

Histological types: ALM - acral lentiginous melanoma, SSM - superficial spreading melanoma, NM - nodular melanoma, LMM - lentigo maligna melanoma.

### Histopathological data

Frozen specimens (snap frozen immediately after surgery) were subjected to this evaluation. In order to relate protein expression data to the tumor cellular composition, histological analyses were performed on the frozen tissue sections adjacent to sections used for mass spectrometry (see Methods). Parameters such as tumor content, surrounding lymph node area, necrosis and connective tissue percentages and lymphocytic infiltration were examined by a certified pathologist (Table 2).

**Table 2:** Tumor and tumor samples properties.

Samples’ properties:	mean	sd	min	max
tumor %	66	33	0	99
necrosis %	5	11	0	63
lymph node %	12	23	0	97
connective tissue %	17	26	0	100
**Tumor properties**		**n**	**%**	
tumor cell size	<*20 microns*	98	88	
	*20–25 microns*	2	2	
	>*25 microns*	1	1	
tumor cell shape	*epithelioid*	82	74	
	*mixed epithelioid and spindle*	17	15	
	*spindle*	2	2	
Tumor cell pigmentation	*0*	48	43	
	*1*	20	18	
	*2*	13	12	
	*3*	20	18	
Lymphocyte density	*0*	17	15	
	*1*	37	33	
	*2*	33	30	
	*3*	11	10	
Lymphocyte distribution	*0*	17	15	
	*1*	35	32	
	*2*	25	23	
	*3*	21	19	
Immunoscore, = sum of lymphocyte density and distribution	*0*	15	14	
	*1*	3	3	
	*2*	24	22	
	*3*	18	16	
	*4*	16	14	
	*5*	16	14	
	*6*	6	5	

Tumor cell pigmentation (0 = absent: no melanin pigment discernible even at high power magnification, 1 = slight: melanin pigmentation hardly visible at low power, at high power, melanocytes show a faint diffuse hue or a few scattered melanin pigment granules, 2 = moderate: pigmentation visible at low power, the cytoplasm is translucent and appears significantly lighter than the hematoxylin stained nuclei, 3 = high: pigmentation is easily visible at low power, the cytoplasmic pigmentation reaches an intensity approximating that of the nucleus).

Lymphocyte distribution (0 = no lymphocytes within the tissue, 1 = lymphocytes present involving <25% of the tissue cross sectional area, 2 = lymphocytes present in 25 to 50% of the tissue, 3 = lymphocytes present in >50% of tissue).

Lymphocyte density (0 = absent, 1 = mild, 2 = moderate, 3 = severe).

**Figure 1:**
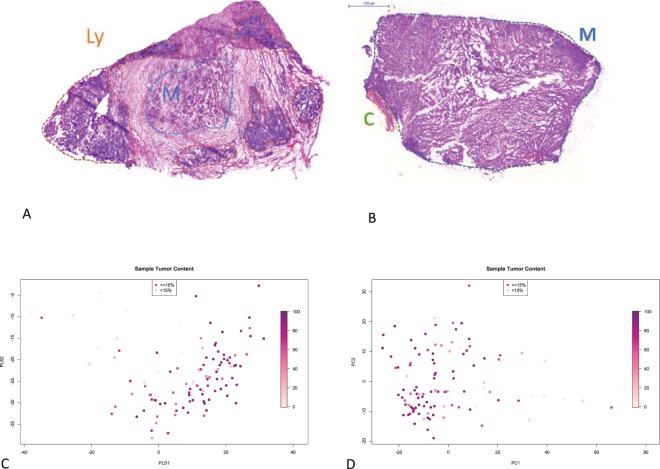
Variability of the tumor samples. (**A**,**B**) Representative histopathology images of the tumor samples. (**A**) Low tumor content sample. Ly – lymphatic cells, M – tumor. (**B**) High tumor content sample. C – connective tissue. (**C**,**D**) Unsupervised multidimensional analysis of the proteomics data. Colouring by tumor content (dark: high content). Samples with <15% tumor shown as triangles, others – as circles. (**C**) Partial Least Squares (PLS) analysis. (**D**) Principal Component Analysis (PCA).

The range of tumor content was 0 to 100%, and for most downstream analyses the inclusion criterion was to have at least 15% neoplasm of the tissue. The pieces for this analysis were removed from the surgically resected sample at macroscopic examination (grossing), thus, their content cannot represent the whole material excised from the patient. Nevertheless, assuming that histopathological properties in lymph node metastases display relatively low variation^17^ we correlated the information with clinicopathological and proteomic data. The samples were mostly composed of epithelioid shaped melanocytes infiltrating the lymph nodes, displaying necrosis to various extent, the background was lymphocytic sheets of otherwise normally appearing lymph nodes, in most cases with connective tissue present (Fig. 1A,B, Table 2).

### Proteomics data

Samples were analysed by high-resolution tandem mass spectrometry. Label-free LC-MS/MS analysis allowed the quantitation of 4963 proteins, and more than one third of them was quantified in more than 50% of samples (see Suppl. Fig. S1). Most analyses of protein expression data, e.g. correlation with tumor content/percentage and patient overall survival, were restricted to 1306 proteins, i.e. those quantified in at least 70% of the samples.

### Relationship of protein expression to tumor content

In this relatively heterogeneous sample set, many proteins exhibited significant correlation to histopathological features. Two hundred and five proteins were significantly positively correlated to sample tumor cell content (using unadjusted p-value < 0.0001) and a smaller number, 29 proteins, were negatively correlated. As expected, the proteins correlated to tumor cell content usually showed inverse correlation to connective tissue content. In principle, correlation p-values should be adjusted for multiple testing using Benjamini-Hochberg (BH) approach. Approximately, the conservative raw p-value of 0.0001 used here corresponds to the value of 0.006 after the BH correction (Suppl. Table ST7).

Positive and negative correlation of protein expression to tumor cell content was connected to particular molecular and biological functions. A Panther^18^ analysis of tumor cell-correlated proteins yielded molecular functions such as tRNA ligases and glycogen phosphorylases for the positively correlated set, while complement activation, structural constituent of cytoskeleton and actin-binding characterized the proteins negatively correlated to tumor content. Similarly, an Ingenuity Pathway Analysis (IPA) performed for the tumor-correlated proteins provided relationship networks enriched in proteins related to transcription, translation, glycolysis, tRNA charging, ubiquitination, tubulins, and splicing (See Suppl. Fig. S2 and Suppl. Table ST1). Similar functional themes were found to be associated with tumor cell content in a smaller subset of the current cohort analysed previously^19^, thus, confirming our earlier pilot findings. These functions are in line with well-known features of malignant tumors and connective tissues, and suggest that proteomics data could be used for tissue discrimination and quality assessment of the sample with respect to tumor content^20^.

### Unsupervised view of the data - PCA

A non-supervised multivariate analysis of proteome profile allows to explore the main components of variability between the melanoma samples. Here, a principal component analysis (PCA) of protein expression data did not show obvious separation with respect to clinical or histopathological parameters (e.g. BRAF mutation status, survival, see Suppl. Fig. S7A,B). The only exception was tumor cell content, where a clear trend was visible (see Fig. 1C,D) indicating that sample heterogeneity in terms of tumor cell content was a major source of variability in the proteomics data.

### Relating proteomics data to survival

**Figure 2:**
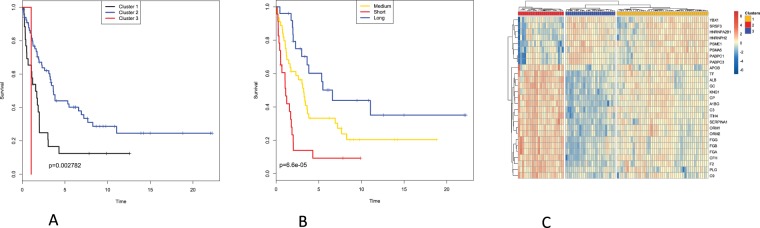
Proteomics data is related to patient survival. (**A**,**B**) 2A. Kaplan Meier plots for patient clusters obtained by (**A**) consensus clustering using 1306 proteins quantified in at least 70% of the samples (shown in Suppl. Fig. S3C) (**B**) consensus clustering using only the 27 survival-related proteins, with significant Cox scores (shown in Suppl. Fig. S3D). (**C**) Two-way hierarchical clustering of the 27 survival-related proteins and the patient samples. Red: high expression. Blue: low expression. Patient clusters coloured as in (**B**).

In order to relate protein expression in lymph node metastatic melanomas to patient survival, we attempted an unsupervised classification based on consensus clustering^21^. This approach, applied to the whole sample set (111 patients) produced clusters that did not differ significantly in survival (Suppl Fig. S3A,B). Thus, for subsequent analyses only the 96 samples with tumor content of at least 15% were considered (choosing higher thresholds did not improve survival prediction while obviously lowered the number of available samples). Here, we investigated the predictive power of the protein expression data from metastatic melanoma using two approaches. The unsupervised approach involved hierarchical consensus clustering. The supervised approach consisted of Partial Least Square (PLS) regression in combination with Cox Proportional Hazards modeling (PLS-Cox). Both approaches were able to produce patient clusters with significant differences in survival. Applying unsupervised clustering to the proteomic data produced three patient clusters which show distinct differences in survival (log-rank test p-value = 0.0028, see Fig. 2A).

The PLS-Cox model reduces the expression of the whole feature-set (~1300 proteins) to a single latent (inferred) variable, which explains the main part of the variability with respect of patient survival and which is then used in a Cox Proportional Hazards model. A high score on this latent variable is linked to a low hazard score, i.e. better prognosis. Furthermore, we used rank products to extract the features (proteins) which contribute most to the latent variable^22^. After cross-validation and FDR testing, we obtained 27 proteins which were strong contributors to the latent variable (see Suppl. Table ST2). Of these, 9 were positively correlated (thus high expression is linked to long survival) and 18 negatively correlated (overexpression of these is linked to short survival).

When applied to only the 27 proteins obtained from the PLS-Cox model, the same hierarchical clustering algorithm gave us three patient clusters, even more distinct in terms of survival (log-rank test p-value = 0.000066, see Fig. 2B). One of the clusters corresponded to poor survival and was characterized by downregulation of the 9 proteins positively correlated and by upregulation of the 18 proteins negatively correlated to survival. A second cluster had expression profiles opposite to those of the first one and corresponded to a more favourable survival. A third cluster corresponded to intermediate survival and an intermediate expression pattern (see Fig. 2C).

Analogous analyses were performed using peptide quantitation data. Here, unsupervised consensus and supervised PLS-Cox clustering also produced clusters significantly differing in survival, albeit with weaker effect.

In order to ascertain that the 15% tumor content cutoff was not too subjective, several other cutoffs were tested (0, 25, 50 and 75% tumor) and the PLS-Cox survival analysis was repeated for each. The 15, 25 and 50% cutoffs produced very similar results in terms of candidate survival biomarker sets (Suppl. Table ST6), albeit the 15% threshold provided the largest number of significant candidates (twenty seven). Also, the 15% cutoff provided the most significant statistical model while the 25% cutoff resulted in a model of similar significance (Suppl. Table ST9).

Further, the Cox survival analysis was performed using several histological features of the samples instead of protein expression data (see Suppl. Table ST8). While some such features (related to cytoplasm features) did show a weak relationship to survival (univariate Cox regression model p-values 0.003–0.03), protein expression clearly outperformed these features in terms of survival prediction. All univariate Cox models built for the 27 candidate proteins were significant and most had p-values below 0.003 (minimum 3*10^−6^, see Suppl. Table ST10). Of note, tumor content was not a significant survival predictor (see Suppl. Table ST8).

**Figure 3:**
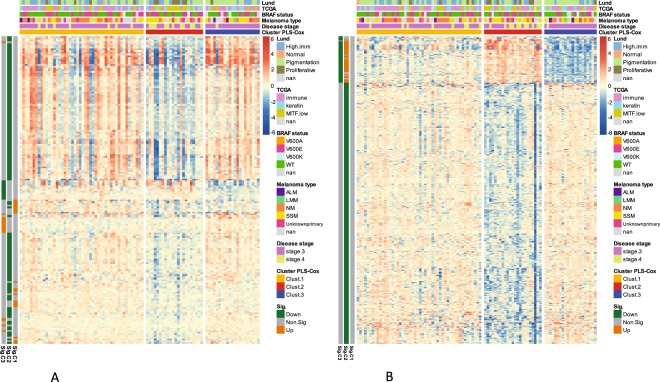
Proteins and mRNA exhibit differential expression among the survival-related patient clusters. Two-way hierarchical clustering of the transcripts (**A**) and proteins (**B**) differentially expressed between the survival-related patient clusters as per SAM analysis. Only highly significant transcripts and proteins shown (q value below 0.0005). Red: high expression. Blue: low expression. Patient clusters coloured as in Fig. 2B. Additional annotations (coloured bars at top) indicate selected patient/sample parameters: Lund genomics cluster^23^, TCGA genomics cluster, BRAF status, Melanoma type, disease stage. Additional annotations (coloured bars on the left, orange or green) indicate that a given transcript or protein is significantly up- or down regulated for a given cluster.

The PLS-Cox based supervised clustering built on protein expression was compared with two genomics-based sample classifications applied previously to the same tumor samples. The four-category classification of Jönsson *et al*. (high immune, normal, pigmentation and proliferative^23^) and TCGA classification (immune, keratin, MITF-low^16^) were not in perfect accordance with the three survival clusters elucidated herein (see Fig. 3 and Suppl. Fig. S4A,B). However, there were clear differences between the longer and shorter survival clusters in terms of composition of the genomics categories. Interestingly, the short survival cluster 2 had largest proportion of proliferative-type tumors (Jönsson’s classification^23^) while the long survival cluster 3 had approx. 75% samples of the pigmentation type. In terms of TCGA classification^16^, short survival cluster 2 had largest proportion of MITF-low tumors while the long and medium survival clusters 1 and 3 had largest proportion of immune-type tumors (Suppl. Fig. S4). The long survival clusters obtained by two approaches (unsupervised and supervised) using protein data agreed well - they were composed mostly of the same patient samples (90% agreement, i.e.: 90% of the samples from the supervised good prognosis cluster belonged also to the unsupervised good prognosis cluster). The same applies to the short survival clusters (78% agreement, see Suppl. Fig. S4C). The chi-squared test comparing the unsupervised and supervised patient sample clustering supports their consistency (p-value < 10^−5^). Interestingly, the short survival cluster (supervised) had significantly higher necrosis content than other clusters (Kruskal-Wallis p-value < 10^−6^, see Suppl. Fig. S6).

Although for the survival prediction model there was no independent proteomics validation cohort available, we performed a tentative validation of the candidate proteomic survival biomarkers found in our study by using a large transcriptomic dataset of melanoma lymph node metastases (TCGA, N = 336, see Materials and Methods). Several of the 27 candidate biomarkers could be validated in this independent cohort, including those positively related to survival (high expression in long survival): PSME1, HNRNPA2B1 and SRSF3, and those negatively related to survival (high expression in short survival): APOB and ORM1 (see Suppl. Table ST5). This result is encouraging, bearing in mind the fact that on the average the corresponding signals for mRNA and protein expression correlate moderately.

### Functional analysis of the survival-related clusters

The three clusters obtained by supervised PLS-Cox analysis^24^ of proteomics data and significantly differing in survival were explored in order to understand the molecular differences. To this end, the current proteomic data and mRNA expression data obtained previously for the same melanoma samples^23^, were subjected to SAM analysis (a technique conceptually similar to ANOVA^25^) to obtain genes and proteins differentially expressed between sample clusters. The analyses included more than 1300 proteins and more than 11000 genes. At significance level of FDR < 0.0005, 419 proteins and 177 genes were found to be differentially expressed between the three clusters (1368 proteins and 777 genes at more relaxed significance level of FDR < 0.05). The heatmaps in Fig. 3A,B show the genes/proteins with cluster-specific expression patterns. Within the three clusters, cluster 3 (long survival) clearly had underrepresentation of melanomas that were stage 4 while cluster 2 (poor survival) clearly had overrepresentation of stage 4 melanomas (see Fig. 3A,B).

The sets of proteins and genes significantly differing between the three survival-related patient clusters were rather different (for FDR < 0.0005, overlap between the 419 proteins and the 177 genes was only 8, while for FDR < 0.05, the gene/protein list overlap was 68, see Suppl. Table ST3). This clearly shows that proteomics and genomics analyses capture to some extent complementary aspects of melanoma biology. Using mRNA profiling data of the same patient cohort (the same tumor samples, but different sections) as previously published^23^, one can correlate mRNA and protein expression signals. For these, a median correlation of 0.306 is obtained (Suppl. Fig. S8). This is generally in agreement with the previous studies, however, since mRNA and protein data were obtained from different tissue sections of the same samples, the actual correlation is probably slightly underestimated.

The differential expression analysis of genes and proteins provides tumor- and survival-related functions in short and long survival sample clusters. Although, the differentially expressed sets of genes and proteins were by large different, the biological functions related to the patient clusters were to a certain extent similar (see Suppl. Table ST4). For the short survival cluster, the significantly downregulated genes and proteins alike were enriched in functions such as antigen processing and presentation, TCR and interferon signalling. The three survival-related patient clusters did not differ in terms of mutation burden in an analysis of genes known to often harbor mutations in melanoma (See Suppl. Fig. S5).

### Functional analysis of the 27 proteins obtained from the PLS-Cox model

**Figure 4:**
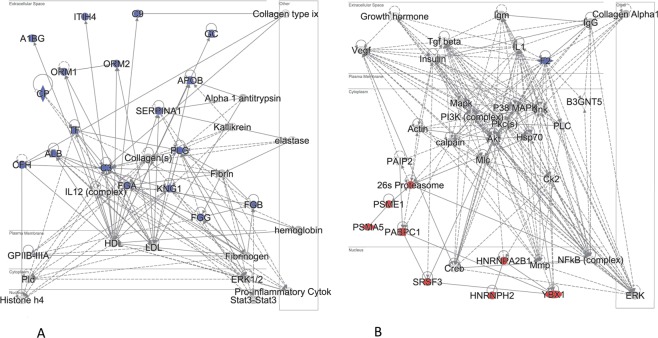
Pathway analysis for 27 survival-related proteins. Ingenuity Pathway Analysis (IPA) for the proteins identified by the PLS-Cox analysis as significantly related to survival (Cox score FDR < 0.1). Protein-protein relationship subnetworks shown that are enriched in the 27 query proteins. (**A**) First subnetwork, (**B**) Second subnetwork. Blue – proteins with expression negatively correlated to survival. Red – positively correlated to survival. Data were analyzed through the use of IPA (QIAGEN Inc., https://www.qiagenbioinformatics.com/products/ingenuitypathway-analysis)^109^.

Ingenuity Pathway Analysis (IPA) split most of the 27 proteins that were guiding the three survival clusters into two functional relationship networks. The first network was mostly extracellular and included proteins negatively correlated to survival (low expression in tumors from patients with good prognosis, i.e. long survival). The second network was a nuclear/cytoplasmic one, and included proteins positively correlated to survival (high in tumors from patients with long survival, Fig. 4A,B). A complementary IPA analysis was executed using an extended set of 160 top proteins most strongly related to survival albeit not all strictly significant. Of these, 80 were negatively correlated to survival and 80 - positively correlated. Here, the proteins positively related to survival as per Cox analysis were enriched in functions such as RNA post-transcriptional modifications, protein synthesis and cell death. The proteins negatively correlated to survival were enriched in cell-to-cell signalling and cell movement proteins.

#### Proteins negatively correlated to survival (high expression in short survival)

Interestingly, many of the 18 proteins showing negative significant correlation to survival are high-abundance plasma proteins. This may reflect the vascularisation aspect of melanoma metastases as well as immune component of tumor development. One might speculate that the lymph nodes are thought to be filters of the circulating lymph which contains enriched fractions of the proteins and lipids of the blood which may show in the results. Alternatively, the tumor cells might be “hiding” while metastasizing and covering themselves with platelets, thus exhibiting expression of platelet proteins (all but one of the 18 proteins are present in platelets^26^). Also, the negative correlation to survival of coagulation-related proteins (F2, PLG, FGB, FGG, FGA, KNG1) likely reflects the well-known relationship between cancer and thrombosis^27^.

The role of the copper and iron transport protein ceruloplasmin (CP) in cancer has been reported^28^ and it was found elevated in plasma of melanoma patients^29^, hence a negative correlation to survival could be expected. Human serum transferrin is a glycoprotein which is involved in iron transport. Since neoplastic cells have a high requirement of iron related to their rate of proliferation^30^, it seems logical that we found high level of transferrin in the poor survival cluster.

More than 5-fold higher level of the protease inhibitor ITIH4 was reported previously in sera from patients with hepatocellular carcinoma with good prognosis compared to patients with poor prognosis^31^. The ITIH4 gene expression was lost in multiple human solid tumors^32^. However, in a rat model for colon cancer, ITIH4 was one of four proteins that was upregulated in sera compared to wild-type rats^33^. The serine protease inhibitor, SERPINA1, has been reported to modulate invasive and metastatic capacity in lung cancer, gastric cancer, and CRC^34–36^. Elevated expression of SERPINA1 was previously correlated with advanced stage, lymph node metastasis, and poor prognosis^37^, which is in accordance to our current findings.

Complement factor H (CFH) is the main actor inhibiting complement responses by regulating the Complement Alternative Pathway^38^. CFH binds to “*self marker*” structures on matrix and the cell surface, e.g. GAG chains and sialylated sugars, and prevents further activation/attack by the complement system^39^. CFH may have dual roles in cancer, either promoting tumor progression (by immune evasion) or supporting tumor suppression (by inducing an anti-inflammatory microenvironment^38^). Tumor cells may “hijack” the complement system by expressing, releasing or recruiting CFH and other complement inhibitors in high amounts, thus evading complement attack. This has been described in ovarian, lung, glioma and colon cancer cells^40–43^. In addition, CFH has been suggested as a marker in lung adenocarcinoma^44^, where shorter survival time of patients with adenocarcinoma was associated with increased CFH staining. Data from the TCGA cohort suggest that increased mRNA levels of CFH are significantly related to poor prognosis in kidney carcinoma^45^ and urine levels of a closely related protein CFHR1 were negatively related to bladder cancer survival^46^. To our knowledge, negative relation of CFH protein to survival in metastatic melanoma tissue has not been reported.

A role of Vitamin D signaling and the activity of Vitamin D binding protein GC (VDBP) in melanoma is known^47,48^ and vitamin D deficiency is associated with worse prognosis^49^. VDBP is responsible for transporting Vitamin D analogues in plasma. While SNPs in VDBP were reported not to influence melanoma survival in a case-control study^50^, meta-analysis of VDBP polymorphisms suggested that VDBP rs12512631 TT genotype was linked to a poorer survival compared with those with TC and CC genotypes^47^. The involvement of VDBP in cancer has a complex mechanism: on one hand, VDBP enhances epithelial ovarian cancer progression^51^, on the other hand, higher circulating VDBP levels were observed in healthier melanoma patients^52^. Also, a meta-analysis including 28 studies of 12 different cancers, and analyzing VDBP protein levels vs. cancer risk found trends toward significance (lower risk related to high expression), suggesting a role of VDBP in cancer etiology^53^. The negative relation of VDBP expression to melanoma survival observed by us is not in agreement with some previous reports, whereas promising results were obtained by using VDBP in cancer immunotherapy^54,55^. However, these results cannot be compared directly with ours since serum levels of VDBP need not be correlated to levels in tumor tissue.

APG1 and 2 (Orosomucoid 1 and 2) are heavily glycosylated acute phase reactants, mainly expressed in the liver but also extrahepatically^56,57^ and increased in the circulation during acute inflammation as well as in several cancers including melanoma^58–60^. APG1 seems to be the primary acute phase responder while the proportion of APG1 to APG2 changes significantly in cancer^59^. The APGs display a multitude of biological activities such as acute-phase reactants, modulating immunity, and maintaining the barrier function of capillaries^56,57^. In addition, APGs are involved in binding synthetic drugs which has been described in cancer patients^61–63^. Aberrant glucosylation of the APGs is related to pathophysiological situations including cancer^64^. Overall, the negative relation to melanoma survival of APGs detected in metastatic melanoma tissue in the current study would be in agreement with previous literature describing circulating levels in cancer patients.

A recent study^65^ related serum albumin levels to melanoma stage in a large patient cohort showing a significant reduction in circulating levels in stage 4 and in older patients. Albumin is a negative acute phase protein, e.g. levels are reduced during inflammation. The reduced levels in cancer and several other illnesses may be due to decreased synthesis, increased catabolism and other mechanisms^66,67^. In the current study, serum albumin level in melanoma tissue is negatively related to survival (high in patients with poor survival) which appears not in accordance with most other studies. However, most studies look at circulating levels and not metastatic tumor tissue.

Apolipoprotein B-100 (APOB) is a receptor for cholesterol which has been shown to increase melanogenesis^68^ and targeting cholesterol transport in melanoma CTCs was shown to retard metastasis development^69^. This may be in line with current results of increased APOB expression in poor survival.

Alpha-1B-glycoprotein is a secreted glycoprotein with some similarity to the immunoglobulin family and basically very few known functions^70^. Interestingly, it has been described in proteomic studies of several cancer types like breast cancer^71^, oral squamous carcinoma^72^, in the serum of non-small cell lung cancer^73^, and in pancreatic ductal adenocarcinoma^74^. Here we describe for the first time a negative correlation of alpha-1B-glycoprotein tissue expression to melanoma survival.

#### Proteins positively correlated to survival (high expression in longer survival)

The splicing factor SRSF3 has been reported as an oncogenic factor in several types of cancer^75–79^. However, in colorectal cancer, loss of SRSF3 was significantly associated with poor survival and shorter disease-free survival in early cancer stages^80^. It was also shown that loss of SRSF3 was necessary for metastatic cells to colonize the liver microenvironment in mice^80^. Loss of SRSF3 has also been shown to predispose to hepatocellular carcinoma^81^ and myeloid leukemia^82^. In this study, higher expression of SRSF3 was also found in the better prognosis cluster.

The transcription factor YBX1 is positively associated with a proliferative cellular state and might therefore be reported to be overexpressed in a variety of human cancers^83–86^. However, the YBX1 expression seems to be tightly regulated by a feedback mechanism ensuring optimal proliferation and survival of melanoma cells. The levels of YBX1 are also critical in melanoma cells for proliferation. High levels inhibit cell cycle progression and low levels induce apoptosis^87^. The YBX1 has been reported to correlate with bad prognosis in liver cancer^88,89^ while here YBX1 is upregulated in melanoma patients with good prognosis.

Among the proteins positively correlated to survival, there are two proteasome related proteins PSMA5 and PSME1. The role of immunoproteasome in cancer is known^90^, however high expression in better prognosis patients is not an obvious result. In a recent meta-analysis, PSMAs were generally found to be upregulated in cancers, including melanoma. Expression of some members of the PSMA family correlated with poor prognosis^91^, however no melanoma prognosis data was available for the PSMA5 gene/protein that we find correlated with better prognosis. The Proteasome activator PSME1 (PA28alpha) that has been reported to regulate presentation of T lymphocyte epitopes on melanoma cells^92^ is found here to be upregulated in good prognosis melanoma patients, similarly to a previous proteomics study^15^. Interestingly, quite to the opposite, in oral squamous cell carcinoma PSME1 expression has been reported to be related to poor prognosis^93^.

The Poly A binding proteins PABPC1 and PABPC3 function in post-transcriptional control of mRNA and regulate cell proliferation^94^. PABPC1 expression was previously reported positively correlated to survival in esophageal cancer^95^, but this protein has also been found to be oncogenic in gastric carcinoma^96^.

The splicing factor HNRNPA2B1 has been reported as a candidate biomarker in lung cancer and regulator of epithelial-mesenchymal transition in pancreatic cancer (PDAC)^97–99^. Another splicing factor, HNRNPH2, was shown to drive anticancer drug resistance^100^ and to drive hepatocellular carcinoma development^101^. Hence, higher expression in good prognosis of these two factors is an unexpected result.

## Conclusion

We present a comprehensive proteomic, histopathological and genomic evaluation of malignant melanoma lymph node metastases. Our study is unique in applying in-depth histopathological characterisation to individual tumor samples. This, combined with detailed clinical information, allows elucidation of an efficient set of proteomic prognostic biomarkers. Since many of these candidate biomarkers are known to be relatively common plasma proteins, they present a possible opportunity for development of prognostic blood-based biomarker panel. This work builds on our own exploratory studies^19,102^ as well as work by other groups^15,103^ but differs from the previous work also by a much larger study cohort. By analysing the protein data alongside the genomic data obtained of the same tumor tissue, we highlight the complementarity of proteomic and transcriptomic molecular images of melanoma.

The fact that some of the prognostic proteins have not been reported in melanoma context before, and the fact that some exhibit unexpected relationship to survival, only exemplifies the complexity of melanoma progression mechanisms.

## Materials and Methods

### Reagents and solutions

All chemical reagents were purchased from Sigma Aldrich (St. Louis, MO) unless otherwise specified. Water and organic solvents were of LC–MS quality and supplied by Merck (Darmstadt, Germany). All solutions were degassed by sonication before use.

### Tissue samples and sample preparation

111 lymph node metastasis samples from patients with malignant melanoma (Stage 3 and 4), archived in the local malignant melanoma biobank were obtained from Skåne University Hospital, Sweden. Each sample was marked as ‘MM’ followed by identification number. Ethical approval was granted by Central Ethical Review board at Lund University; approval number: DNR 191/2007, 101/2013. All patients within the study provided a written informed consent. All experiments were performed in accordance with relevant guidelines and regulations. The malignant melanoma biobank “Tissue bank for research on tumour diseases” (BD20)” is located at Barngatan 2B, 221 85 Lund, Sweden. The samples were originally snap frozen immediately after surgery. Frozen tissue samples from BD20 were sectioned on a cryostat into 10 µm thick slices (approximately 6 × 6 mm), placed into a 96 well plate and stored at −80 °C until further use. From each tissue, 15 to 20 slices were withdrawn for sample preparation. Patient characteristics are summarised in Table 1. Clinical and histopathological parameters were retrieved from patients’ clinical records, pathology reports and the Swedish National Population Registry. Survival was defined as time (days) from lymph node excision to patient’s death or censoring date.

### Histopathological evaluation

Frozen sections of all lymph node metastases stained with HE were evaluated by a certified pathologist. Serial sections were taken of each tumor, and at least seven slices per sample were examined. The tissue was assessed for its content regarding tumor, normal lymph node, necrosis, and background of any further component (e.g. fat or connective tissue). As previously described^16,19^, the tumor was then evaluated for its histological characteristics containing epithelioid or spindle or mixed architecture, the tumor cell average size (scale 1–3) and pigmentation (scale 1–3). The tumor infiltrating lymphocytes were also assessed for their distribution (scale 1–3) and intensity (scale 1–3) in the tumor - only those which directly infiltrated the metastases were taken into account. The sum of distribution and density was then summarized in a 0–6 score considered as immunoscore.

### cDNA synthesis and BRAF DNA sequencing

Two cell lines, SK-MEL-2 and SK-MEL-28 (ATCC^®^, Manassas, USA), were used as reference BRAF wild type and V600E respectively. Total RNA was extracted from the cell lines or frozen tissues from the malignant melanoma patients using RNeasy mini kit (Qiagen, Venlo, The Netherlands). The extracted RNA were reverse transcribed to cDNA by using Superscript III First Strand Synthesis System kit (ThermoFisher, Waltham, MA) according to the manufacturer’s instructions. The cDNA was amplified with a set of primers that produced a PCR product including BRAF mutation at the position V600; 5′-(AGCCTTACAGAAATCTCCAGGACC)-3′ and 5′-(TTGGGGAAAGAGTGGTCTCTCATC)-3′. The PCR conditions were 95 °C for 5 min, followed by 36 cycles of 95 °C for 30 sec, 62 °C for 30 sec, and 72 °C for 2.5 min with a final incubation of 72 °C for 7 min. A portion of the PCR product was amplified a second time using the same condition as the first PCR, and the amplification was 24 cycles, instead of 36 cycles. The PCR products were run on a 1% agarose gel, and DNA was extracted from the gel using a QIAquick Gel Extraction kit (Qiagen) according to the manufacturer’s instruction. The purified PCR products were sequenced using a primer 5′-(TTCCACAAAGCCACAACTGG)-3′ by Eurofins Genomics (Ebersberg, Germany).

### Mutation data

Mutational information for selected 1697 cancer-associated genes were obtained by targeted deep sequencing of the patient tumor samples with matched blood, as described previously^23,104^. Visualization of mutational information was obtained using the oncoprinter function from R package ComplexHeatmap^105^.

### Tissue lysis and protein extraction

Frozen tissue slices were lysed with 6 M urea in 50 mM ammonium bicarbonate buffer (AmBic) for 30 min on ice bath. Samples were additionally vortexed for 10 min in order to promote protein extraction. After incubation with urea the lysate was sonicated for 5 min and centrifuged at 10 000 g at room temperature for 10 minutes. Supernatant was transferred into a new tube and the pellet was discarded. Protein concentration was measured using a bicinchoninic acid protein assay according to the manufacturer’s instructions (Micro BCA kit, Pierce/Thermo Scientific, Rockford, IL). The samples were spiked with 0.1 mg of internal standard – chicken lysozyme (CL, Swiss-Prot accession no. P00698).

### In-solution digestion with trypsin

A fixed amount (80 μg) of protein were reduced with 10 mM DTT for 1 h at 37 °C, then it was alkylated using 50 mM iodoacetamide for 30 min and kept in dark at room temperature. Urea was removed from the samples using Amicon Ultra centrifugal filters (0.5 mL, 10 kDa, Millipore, Ireland) according to the manufacturer’s instructions. Briefly, the protein samples were mixed with 200 μL of 50 mM AmBic, then centrifuged at 14 000 g at room temperature for 20 minutes and the eluates were discarded. These steps were repeated two more times. The samples were transferred to an Eppendorf tube and digested with sequencing grade trypsin (Promega, Madison, WI) in a ratio 1:100 w/w (trypsin:protein) overnight at 37 °C. The digestion was stopped by adding formic acid till 1% as final concentration. The samples were dried using a centrifugal evaporator and resuspended in 80 μL of 0.1% formic acid and centrifuged for 5 min at 10 000 g. The supernatants were stored at −80 °C until further use. Prior to injection onto LC–MS/MS, 20 µL of samples were mixed with 20 µL of peptide retention time calibration mixture (PRTC, Pierce/Thermo Scientific, Rockford, IL, 20 fmoL/mL).

### LC-MS/MS Analysis of the tumor lysate digests

Online chromatography was performed with a Thermo Easy nLC 1000 system (Thermo Fisher Scientific) coupled online to a Q-Exactive Plus mass spectrometer (Thermo Scientific, San José, CA). The peptides were first loaded onto a trap column (Acclaim1 PepMap 100 pre-column, 75 µm, 2 cm, C18, 3 mm, 100 Å, Thermo Scientific, San José, CA) and then separated on an analytical column (EASY-Spray column, 25 cm, 75 µm ID, PepMap RSLC C18, 2 mm, 100 Å, Thermo Scientific, San José, CA). Flow rate of 300 nL/min and a column temperature of 35 °C were utilised. A gradient was applied, using solvent A (0.1% formic acid) and solvent B (0.1% formic acid in acetonitrile). The gradient went from 5% to 40% B in the first 120 min, followed by raise to 90% B in the next 5 min, which was maintained for 10 min. To avoid carryover, each sample analysis was followed by a blank injection (water containing 0.1% formic acid). Mass spectrometry data were measured using a data-dependent top-15 method. Full MS scans were acquired over m/z 350–1800 range with resolution of 70 000 (at m/z 200), target AGC value of 1∙10^6^ and maximum injection time of 100 ms. Selected ions were fragmented in the HCD collision cell with normalised collision energy of 30%, and tandem mass spectra were acquired in the Orbitrap mass analyzer with resolution of 17 500 (at m/z 200), target AGC value of 1∙10^6^ and maximum injection time of 120 ms. The ion selection threshold was set to 4.2∙10^4^ and dynamic exclusion was 20 s.

### Proteomics data analysis

The LC-MS/MS raw files were analyzed with Proteome Discoverer 2.1 (Thermo Scientific, San José, CA) for protein identification and quantitation. The files were searched against the UniProtKB human database (released May 2016) excluding isoforms. The search was performed with the following parameters: carbamidomethylation as static modification, oxidation of methionine as dynamic modification, 20 ppm precursor tolerance and 0.02 Da fragment tolerance. Up to two missed cleavages for tryptic peptides was allowed. Filters: high confidence at peptides and protein levels were applied (FDR 0.01). Protein intensities were log_2_ transformed, followed by sample median subtraction using R (version 2.41–3).

### Multivariate survival analysis

We have used unsupervised and supervised approaches to linking proteomic data to survival. The unsupervised method was performed using consensus clustering in R with ConsensusClusterPlus library (version 1.42.0). The supervised approach is based on PLS-Cox regression similar to that of Nguyen and Rocke^24^. The PLS-step of the model is used to reduce the high dimensionality of the proteomic data, while Cox regression was used on the first PLS component. We use a similar approach as Bair *et al*.^22^ to assess the performance of this model. For cross-validation, the dataset is split into two subsets; the first is used to fit the model, the second to evaluate its performance. This process is repeated 100 times and the results of all iterations are averaged. Simultaneously, we extract the most important features, i.e. proteins, using rank products^24,106^ of the PLS loadings. Correction for multiple testing with Benjamini-Hochberg approach results in 9 proteins which are significantly positively correlated to long survival and 18 which are significantly negatively correlated at adjusted significance level of 0.05. We performed this analysis both for the full sample set (N = 111) as well as for a subset (N = 94) wherein all samples contain at least 15% tumor. The supervised survival analysis was performed using peptide data as well, but the identified sample clusters showed less significant relationships to survival.

For the Kaplan-Meier survival analysis, the *survdiff* function in R (version 2.41–3) was used, which implements the log-rank test.

Differentially expressed genes and proteins for the survival-related patient clusters were elucidated using the SAM method^25^, applying multiple testing correction as described^107^. Gene expression data for the patient samples analysed in the current study were obtained in a previous study using the same sample set but different tissue sections^23^.

By using the pheatmap library in R, two clustered heatmaps were built for the differentially expressed proteins and genes obtained from SAM analysis (FDR < 0.0005). Melanoma type, disease stage, BRAF status, TCGA classification and four-category classification of Jönsson *et al*.^23^ were used as annotation terms. Comparison of clinical and histopathological parameters between the sample clusters was performed by chi-squared test (categorical variables) and by Kruskal-Wallis test (quantitative nonparametric variables). Differences were considered significant when p-value < 0.05 (without multiple testing adjustment).

The transcriptomic dataset of melanoma lymph node metastases from the TCGA database^16^ was used for validation of the candidate proteomic survival biomarkers found in our study. The SurvExpress tool^108^ was applied to assess if query transcripts were promising predictors of survival.

### Protein set functional analysis

Functional analysis of the protein sets identified with PLS-Cox regression and correlation analysis with tumor content was conducted using IPA, Ingenuity Pathway Analysis (Qiagen, Redwood City, CA, USA)^109^, in particular by generating networks of protein-protein functional relationships. As background, the set of proteins detected in >70% of the samples was used.

Functional analysis of lists of proteins mentioned above was also performed using the Panther server^110^. Overrepresentation of specific functional annotations within the protein lists was determined by Fisher’s exact test, the background protein set consisted of all proteins detected. Gene Ontology annotations, SwissProt keywords, and Reactome and KEGG pathways were used as annotation terms for the enrichment analysis.

## Supplementary information

Supplementary Figures

Supplementary Tables

## Data Availability

The proteomics dataset associated with the current article is publicly available in ProteomeXchange (http://www.proteomexchange.org/), dataset identifier: PXD009630.
