# Supplementary material for: Improved survival prognostication of node-positive malignant melanoma patients utilizing shotgun proteomics guided by histopathological characterization and genomic data
Source: Sci Rep. 2019 Mar 26;9:5154. doi: 10.1038/s41598-019-41625-z (PMC6435712; doi:10.1038/s41598-019-41625-z)
Supplement: Supplementary file 1 — Supplementary Figures [file 41598_2019_41625_MOESM1_ESM.pdf]

Supplementary Material for

Improved survival prognostication of node-positive malignant melanoma patients utilizing shotgun proteomics guided by histopathological characterization and genomic data

Lazaro Hiram Betancourt, Krzysztof Pawłowski, Jonatan Eriksson, A. Marcell Szasz, Shamik Mitra, Indira Pla, Charlotte Welinder, Henrik Ekedahl, Per Broberg, Roger Appelqvist, Maria Yakovleva, Yutaka Sugihara, Kenichi Miharada, Christian Ingvar, Lotta Lundgren, Bo Baldetorp, Håkan Olsson, Melinda Rezeli, Elisabet Wieslander, Peter Horvatovich, Johan Malm, Göran Jönsson, György Marko-Varga

Includes Supplementary Figures S1-S8.
Supplementary Tables provided in separate spreadsheet file.

Suppl. Fig. S1

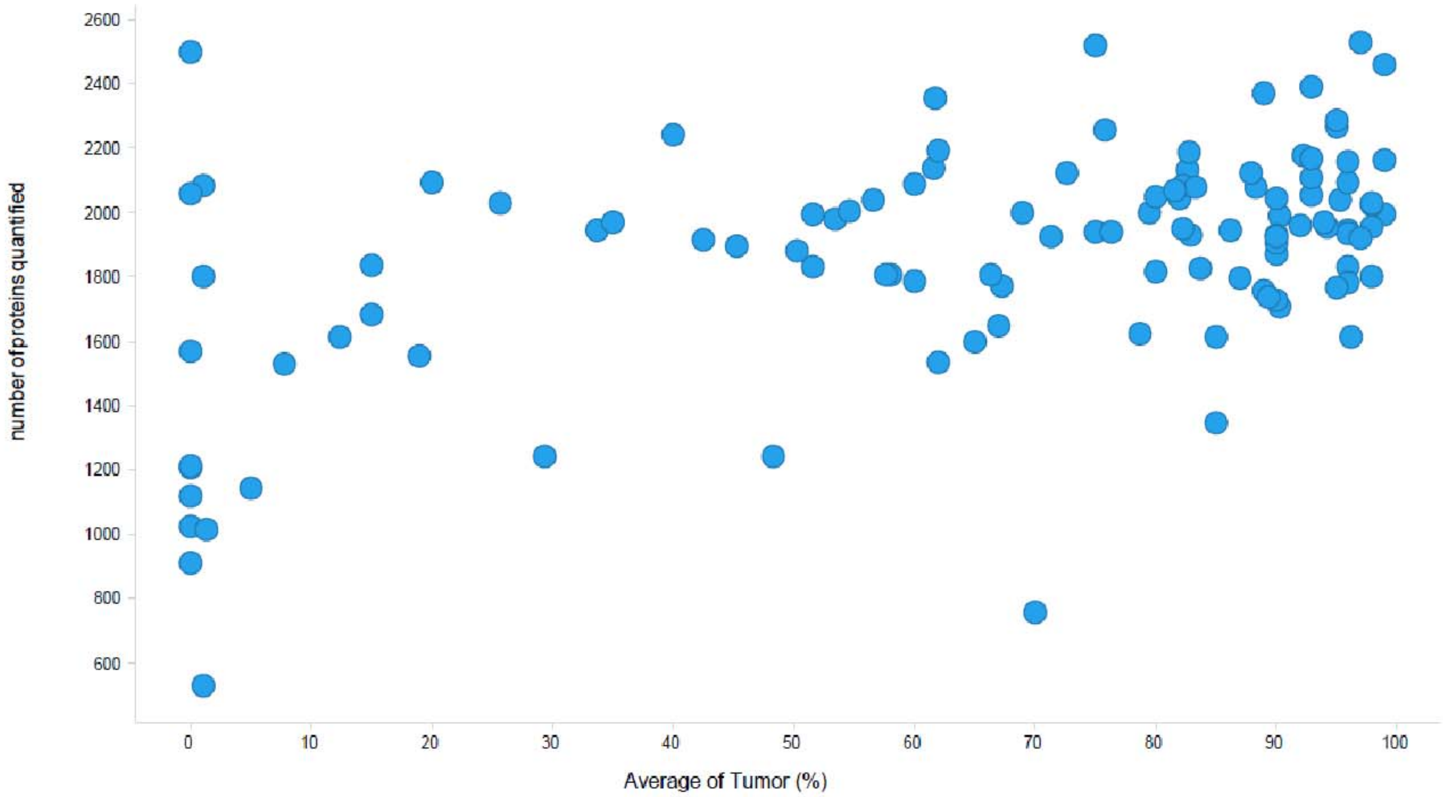

Number of proteins quantified in the sample
vs tumor content of the sample

Suppl. Fig. S2

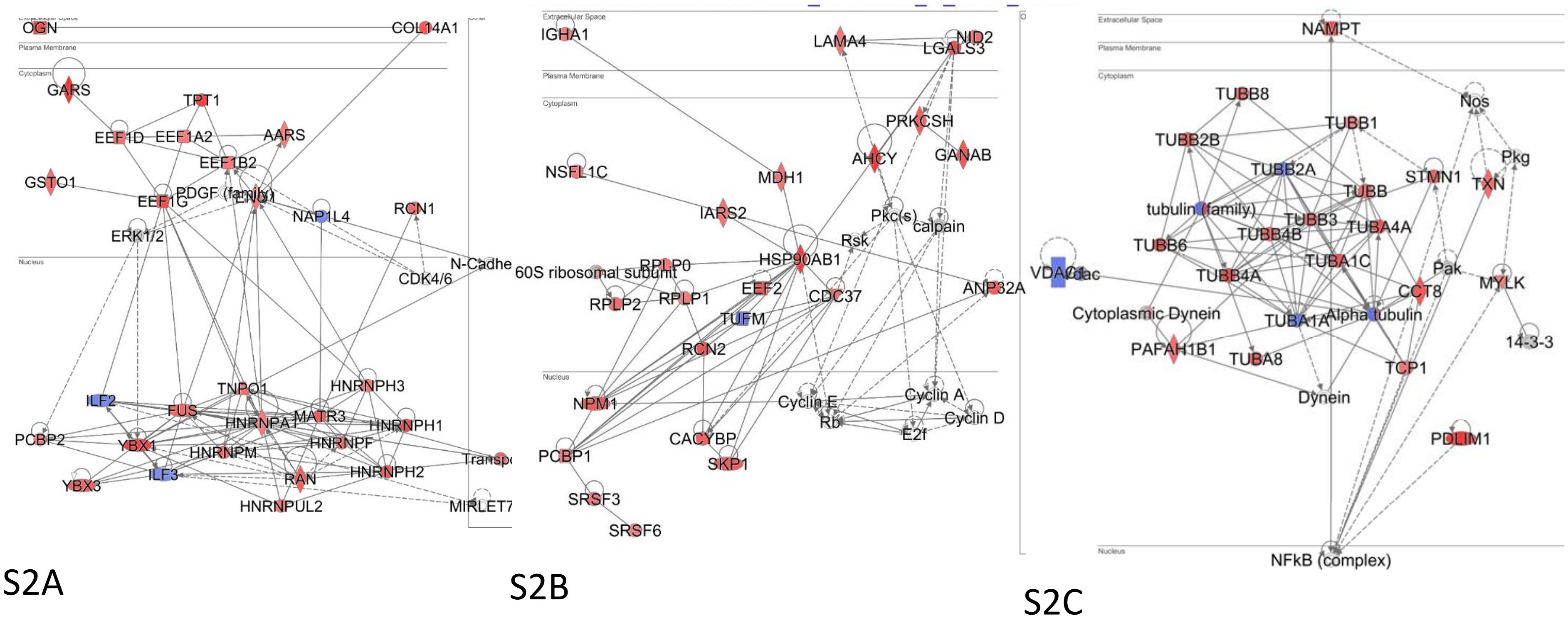

S2A S2B S2C

IPA analysis. Proteins significantly correlated to tumor content; pvalue < 0.0001
S2A. First subnetwork, S2B. Second subnetwork. S2C. Third subnetwork.
Red – proteins correlated to tumor content, blue – anticorrelated proteins.
The networks were generated through the use of IPA (QIAGEN Inc.).

Suppl. Fig. S3A, S3B

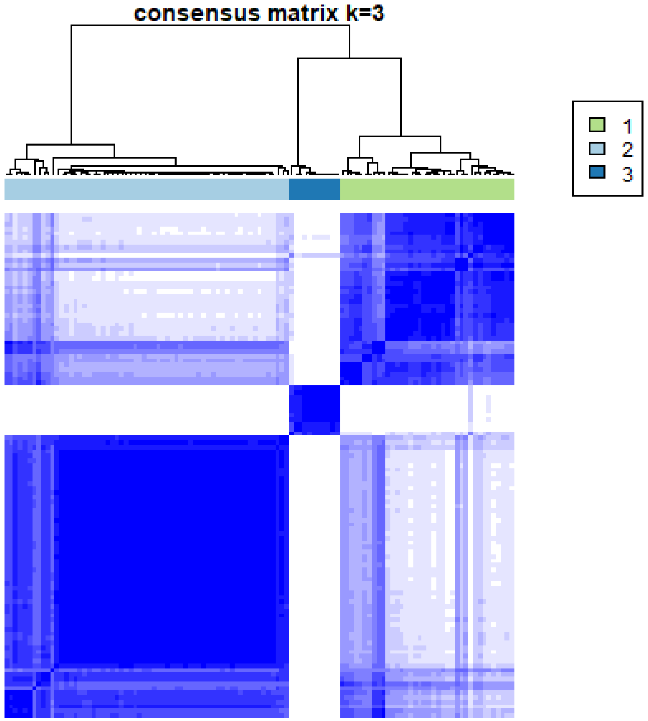

S3A

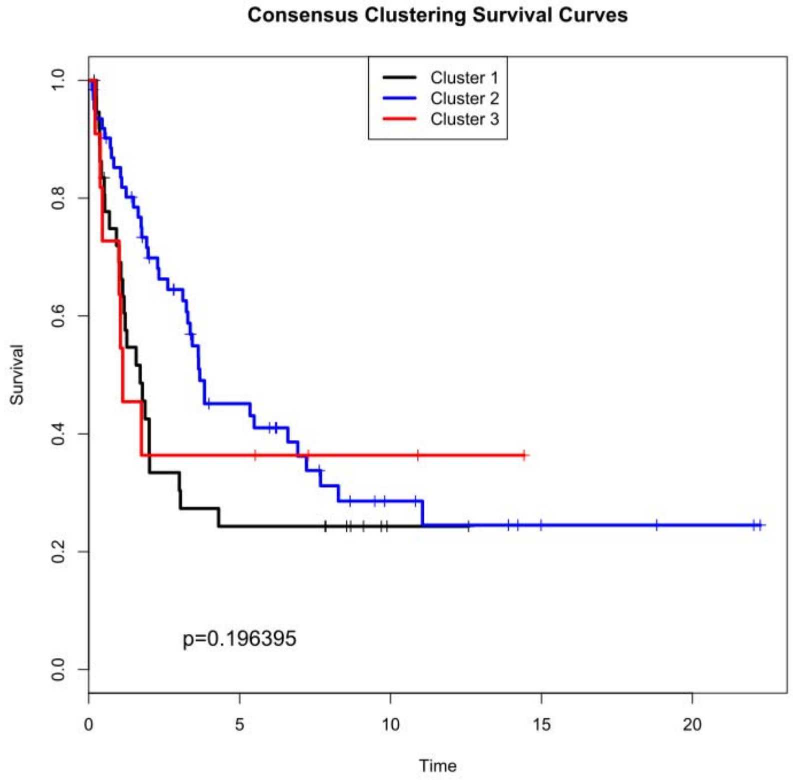

S3B

S3A. Unsupervised consensus clustering, all 111 samples.
S3B. Kaplan Meier plot for patient clusters from S3A.

Suppl. Fig. S3C, S3D

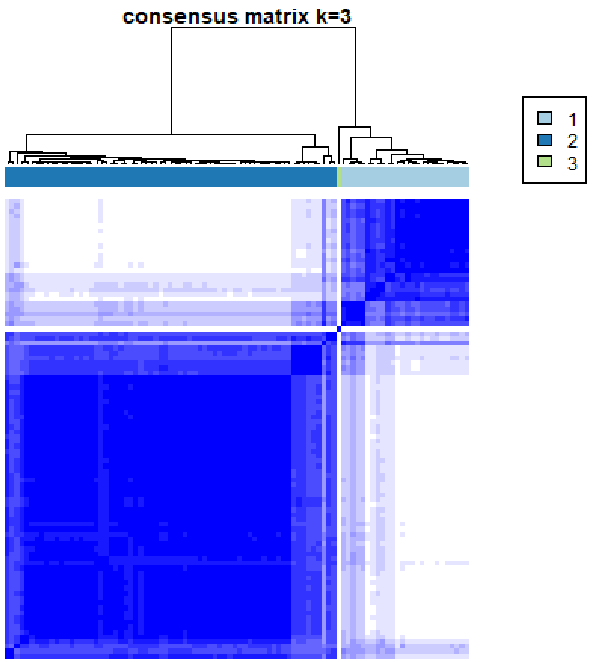

S3C

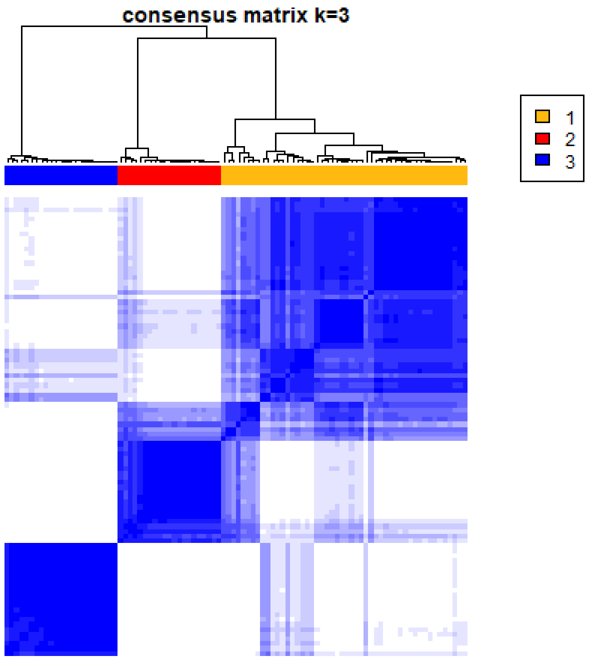

S3D

S3C. Unsupervised consensus clustering, samples > 15% tumor.
S3D. Supervised consensus clustering, samples as in S3C,
but using only 27 proteins with significant Cox scores.

Suppl. Fig. S4

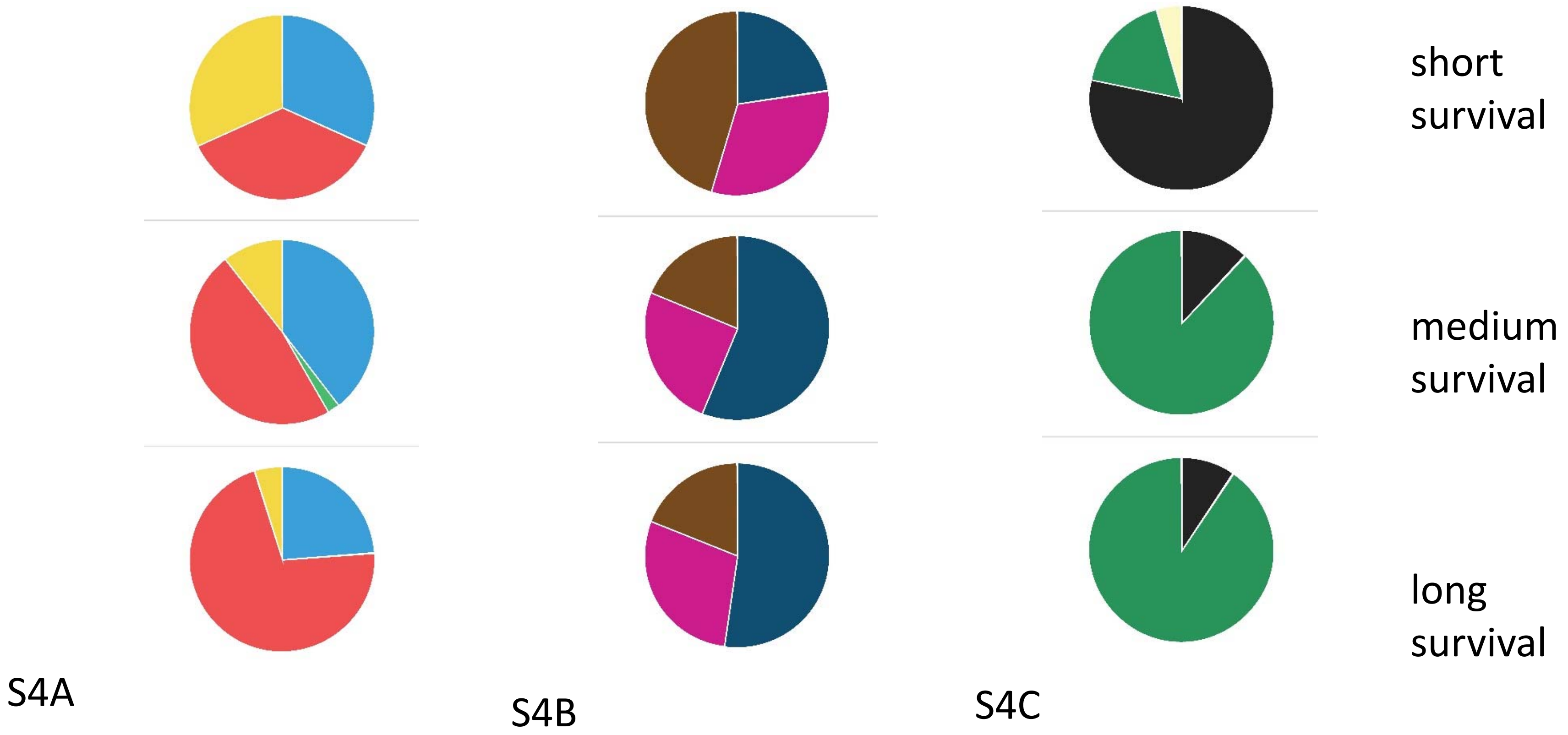

Composition of proteomics-based supervised consensus clusters in terms of genomics-based clusters (S4A, S4B) and unsupervised proteomics clusters (S4C)

S4A, acc. to Jönsson et al. (Cirenajwis, 2015). Blue – high immune, red – pigmentation, yellow – proliferative, green – normal. S4B, acc. to TCGA. Dark blue – immune, brown – MITF low, magenta – keratin.
S4C, unsupervised proteomics clustering. Pale yellow – shortest survival, black – short survival, dark green – long survival

Suppl. Fig. S5

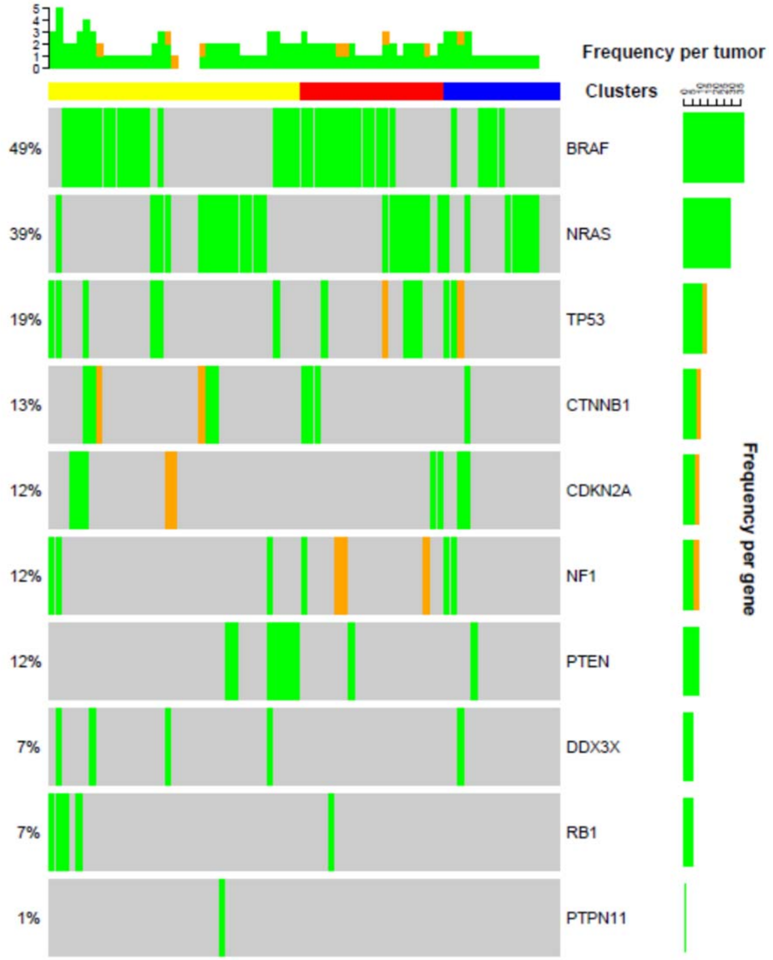

Presence of single nucleotide mutations in selected melanoma-related genes in the survival-related clusters. Mutations: Green – non-synonymous, orange – synonymous. Clusters: Yellow – medium survival, red – short survival, blue – long survival.

Suppl. Fig. S6

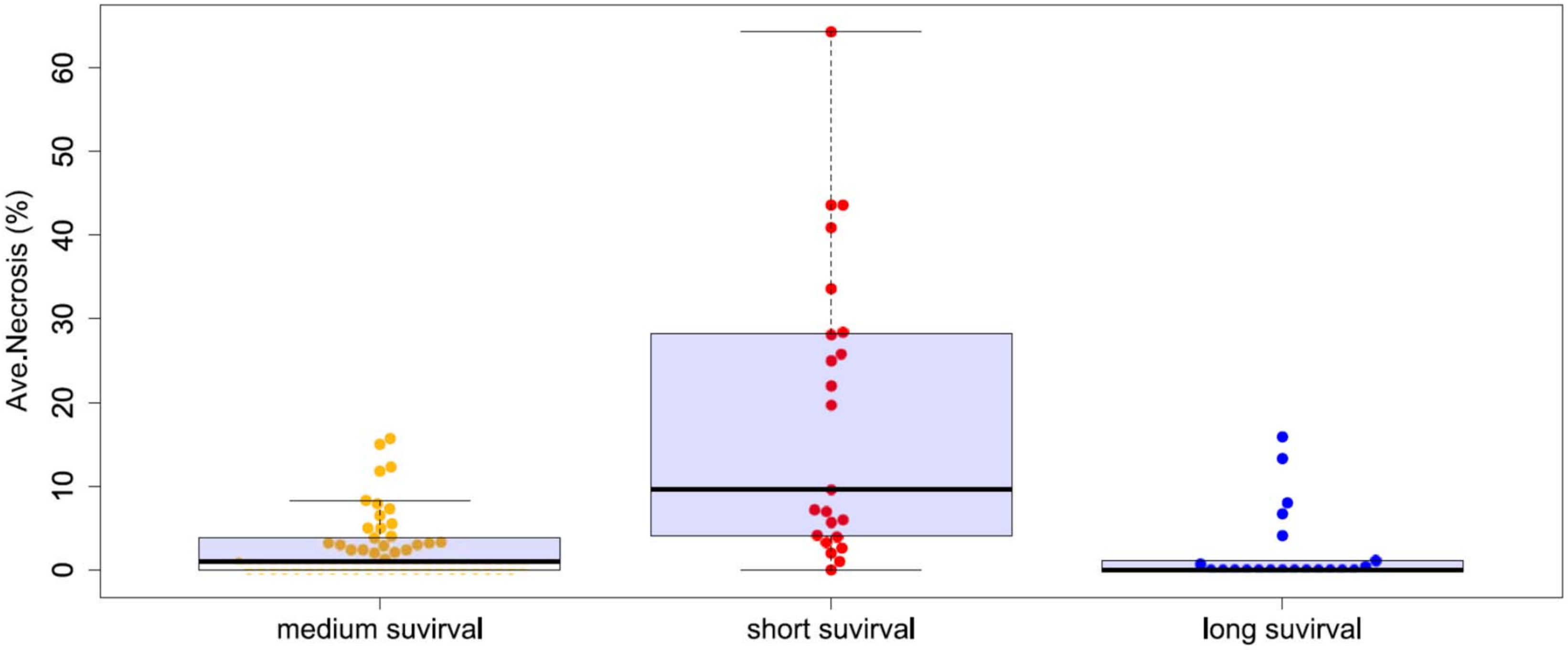

Percentage of necrosis in tumor samples in the survival-related clusters.
The boxplot shows the median, 1st and 3rd quartiles. Observations shown outside the whiskers are considered outliers.

Suppl. Fig. S7

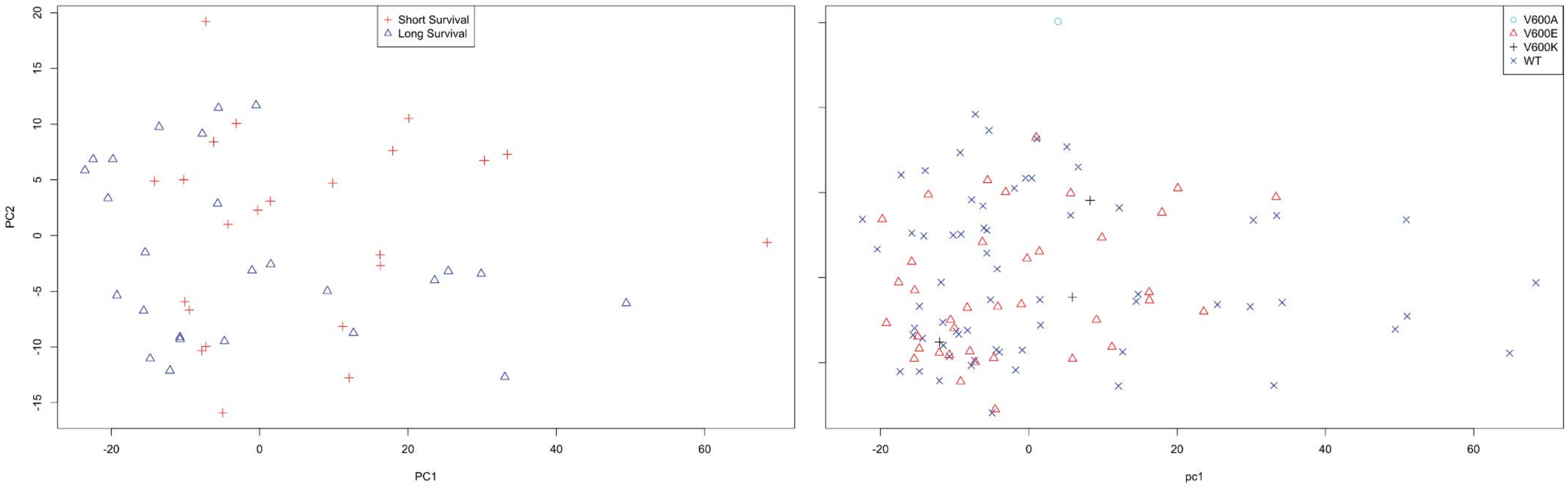

S7A

S7B

A) Principal component analysis (PCA) of the proteomics data with survival information. Short survivors (died within 1 year of sample collection, n = 23) and long survivors (lived at least 7 years from sample collection, n = 26) plotted on the first two principal components.
B) Principal component analysis (PCA) of the proteomics data with BRAF mutation status. The BRAF mutation status of the patients plotted on the two first principal components.

Suppl. Fig. S8

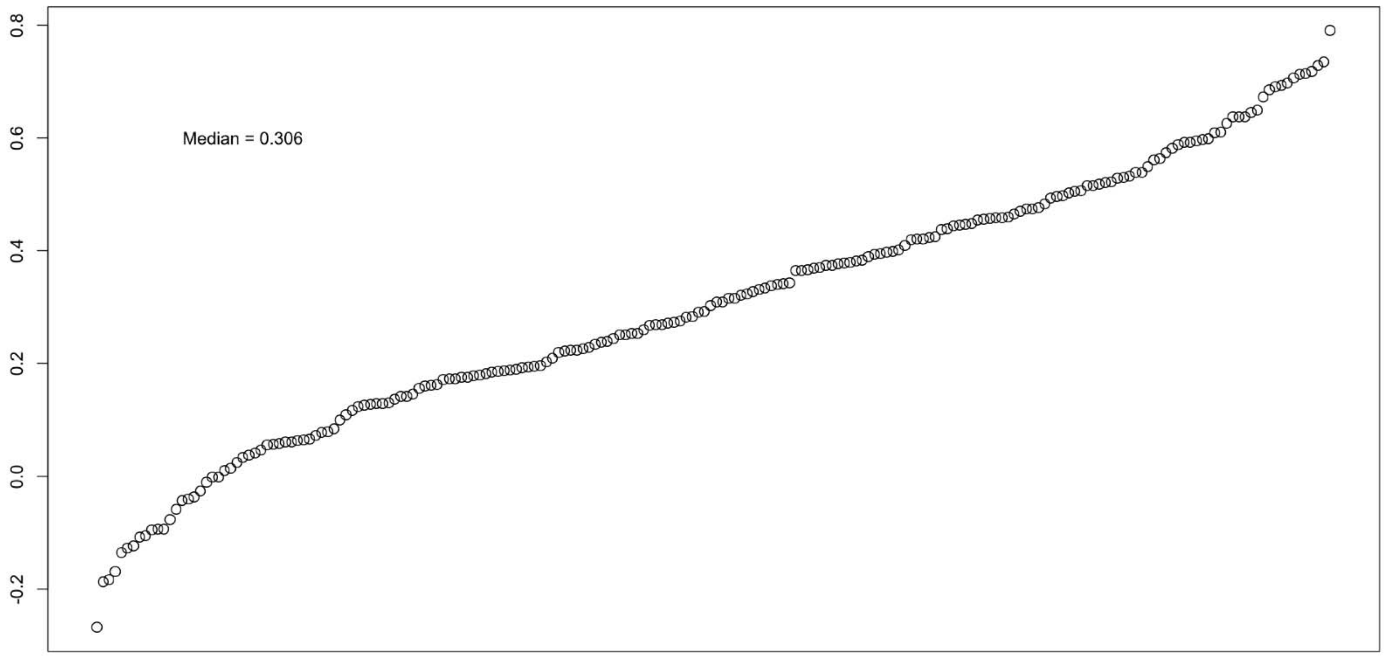

Correlations for corresponding mRNA-protein pairs.
Correlations for the 204 mRNA-protein pairs for which both mRNA and protein data was available and the proteins were quantified in at least 50% of the samples.
